# Hyperoside ameliorates periodontitis in rats by promoting osteogenic differentiation of BMSCs via activation of the NF‐κB pathway

**DOI:** 10.1002/2211-5463.12937

**Published:** 2020-08-18

**Authors:** Tao Xu, Xiao Wu, Zhou Zhou, Yu Ye, Chaoting Yan, Nanshan Zhuge, Jinhua Yu

**Affiliations:** ^1^ Institute of Stomatology Nanjing Medical University Nanjing China; ^2^ Department of Stomatology Central Hospital of Nanjing Nanjing China; ^3^ Key Laboratory of Oral Diseases of Jiangsu Province Stomatological Institute Nanjing Medical University Nanjing China; ^4^ Taian Stomatology Hospital Taian China; ^5^ Department of Endodontics School of Stomatology Nanjing Medical University Nanjing China

**Keywords:** hyperoside, NF‐κB signaling pathway, osteogenic differentiation, periodontitis, proliferation, rat mesenchymal stem cells

## Abstract

Hyperoside, as an active compound, widely exists in a large number of Chinese herbal medicines and has been reported to possess anti‐inflammatory and diuretic properties. However, the effects and underlying mechanisms of hyperoside on periodontitis have not been previously reported. In this study, we found that hyperoside ameliorates symptoms of periodontitis in a rat model, with improvements in alveolar bone resorption, relief of inflammatory infiltration, increase in orderly arrangement of collagen fibers and increase of osteogenic differentiation. In addition, hyperoside promoted proliferation, up‐regulated EdU‐positive cells, decreased cell‐cycle distribution and increased the protein expression of Ki67 and PCNA in rat bone mesenchymal stem cells (rBMSCs), as revealed by Cell Counting Kit‐8, EdU, flow cytometry and western blot analysis. Moreover, hyperoside significantly promoted osteogenic differentiation, as shown by quantitative RT‐PCR, western blot and alizarin red staining assays. Furthermore, hyperoside activated the nuclear factor‐κB (NF‐κB) signaling pathway in rBMSCs, similar to the results observed *in vivo*. Finally, BMS345541, an inhibitor of the NF‐κB signaling pathway, could reverse the effects of hyperoside on the biological functions in rBMSCs. In conclusion, our results suggest that hyperoside has potential therapeutic properties against periodontitis via promotion of proliferation and osteogenic differentiation of rBMSCs via activation of the NF‐κB signaling pathway.

## Graphical abstract

Hyperoside has been reported to possess anti‐inflammatory properties. Here, we confirmed that hyperoside exhibits potential therapeutic properties against periodontitis via promotion of proliferation and osteogenic differentiation of rat bone mesenchymal stem cells through activation of the NF‐κB signaling pathway. Therefore, our study provides evidence that hyperoside may have potential as a novel therapeutic option for periodontitis treatment.

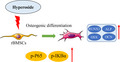

**Abbreviations**
ALP: alkaline phosphatase
BMSC: bone mesenchymal stem cell
CCK‐8: Cell Counting Kit‐8
HGF: human gingival fibroblast
IL‐17: interleukin‐17
LPS: lipopolysaccharide
NF‐κB: nuclear factor‐κB
rBMSC: rat bone mesenchymal stem cell
SD: Sprague Dawley
TNF‐α: tumor necrosis factor‐α

Periodontitis is one of the most common chronic inflammatory diseases and is caused by pathogenic bacteria on the tooth surface characterized with a series of symptoms, including gingival bleeding, periodontal bag, alveolar bone absorption and even tooth loss [1, 2]. As is well known, the local alveolar bone damage caused by the excessive activation of osteoclasts around the roots is closely related to the occurrence of periodontitis, which is one of the obvious histopathological signs of periodontitis [3, 4, 5]. Therefore, more and more researchers have paid attention to the damage of bone destruction caused by periodontitis.

In recent years, with the development of regenerative medicine and tissue engineering technology, the focus of periodontitis treatment has shifted from the control of soft tissue inflammation to the control of pathological bone loss, so as to achieve periodontal tissue regeneration [6, 7, 8]. At present, a variety of regeneration methods have been proposed to treat periodontitis, including guided tissue regeneration, application of platelet‐rich plasma, enamel matrix derivative and so on [9, 10, 11]. However, most of these treatment methods have shown variable and unpredictable clinical results. In addition, factors that affect the inflammatory process, such as poor oral hygiene, extraoral infection and systemic diseases, especially osteoporosis, diabetes and cardiovascular diseases, may be related to this high degree of variability [12, 13, 14]. Bone mesenchymal stem cells (BMSCs) are a kind of stem cell that can differentiate in many directions and can self‐renew and differentiate into various cell types necessary for tissue regeneration [15, 16]. Recent studies have also reported the effects of BMSCs on reducing inflammatory response and producing cytokines related to tissue regeneration. When exposed to an inflammatory microenvironment, exogenous BMSCs can promote tissue formation by self‐differentiation or activation of endogenous progenitor cells [17]. Nevertheless, BMSCs also can be affected by the inflammatory process. Inflammatory or immune‐related cytokines, such as tumor necrosis factor‐α (TNF‐α), interferon‐γ and interleukin‐17 (IL‐17), have been reported to affect the differentiation and induce apoptosis of BMSCs [18]. Therefore, it is of great clinical significance to clarify the role and mechanisms of BMSCs in periodontal tissue regeneration.

Recent studies have demonstrated that the nuclear factor‐κB (NF‐κB) signaling pathway, as one of the main signal transduction pathways, exhibited an essential role in the development and progress of periodontitis [19]. Without external stimulation, NF‐κB binds to inhibitory factor IκB and exists in the cytoplasm in an inactive state. When host cells are stimulated, the upstream kinase IKK of IκB is activated, further phosphorylating, ubiquitinating and finally degrading IκB. NF‐κB, without IκB inhibition, is activated and rapidly nuclear translocated to participate in the progression of periodontitis [20]. It has been reported that TLR4/MyD88 can activate the NF‐κB signaling pathway and induce the secretion of IL‐6, IL‐8, inducible nitric oxide synthase (iNos) and cyclooxygenase‐2, leading to periodontitis [21]. In addition, periodontal pathogens, such as fusobacterium and actinomycetes, can stimulate the NF‐κB signaling pathway of macrophages to induce the expressions of TNF‐α and IL‐6, whereas the NF‐κB‐specific blocker can obviously inhibit this reaction process [22]. Therefore, the NF‐κB signaling pathway plays an important role in the pathogenesis of periodontitis. Nowadays, activation of the NF‐κB signaling pathway by gene intervention or small‐molecule drugs to activate downstream multiple effectors has become a new target of periodontitis therapy [23].

In recent years, the application of Chinese herbal medicine in the treatment of periodontitis has attracted the attention of numerous investigators because of its wide source, few side effects and low price [24]. A great quantity of studies has confirmed that many Chinese herbal medicines have biological effects, such as inhibiting the release of various inflammatory factors, such as IL‐13 and TNF‐α, regulating the differentiation of osteoclasts, adjusting the immune activity of the host and so on [25]. The effective ingredients with positive activities extracted from Chinese herbal medicine may become a candidate drug for the treatment of periodontitis [26]. Hyperoside, as an active compound, widely exists in many Chinese herbal medicines and belongs to flavonol glycosides, with physiological activities such as anti‐inflammation, softening blood vessels, diuresis and so on [27, 28, 29]. However, the detailed role and possible mechanisms of hyperoside in the treatment of periodontitis have not been clarified yet. Therefore, this study was designed to investigate the role and possible mechanisms of hyperoside in periodontitis, and we found that hyperoside could ameliorate periodontitis, which may be achieved by promoting osteogenic differentiation of BMSCs through activating the NF‐κB signaling pathway. Our results provide new insights into the biological functions and underlying mechanisms of hyperoside in periodontitis and will give a novel therapeutic target for treating periodontitis.

## Materials and methods

### Experimental animals

A total of 24 male Sprague Dawley (SD) rats weighing 200 ± 20 g were purchased from the Animal Center of Nanjing Medical University (Nanjing, Jiangsu province, China), and all animals were housed under standard environmental conditions at controlled temperature (22 ± 2 °C), humidity (50 ± 10%) and light (12 h light–dark cycle) with free access to standard diet and water. All procedures for animal care and use were in accordance with the National Institutes of Health guidelines and approved by the Institutional Animal Care and Use Committee of Nanjing Medical University (1801012).

### Establishment of the periodontitis model in rats

The periodontitis model was established following the protocols described previously [30]. Twenty‐four SD rats were randomly divided into two groups of 12 rats each (periodontitis group and periodontitis + hyperoside group). First, 24 male SD rats were anesthetized by intraperitoneal injection of 7% chloral hydrate at a dose of 3 mg·kg^−1^ according to body weight. Then SD rats laid on their back, and their limbs were fixed. Iodophor was used to disinfect the area of maxillary anterior teeth, and orthodontic ligation wire (0.2 mm) was used to wrap the upper anterior teeth. Furthermore, 30 μL lipopolysaccharide (LPS) solution was injected into the ligated gingiva, and then the injection site was pressed with sterile cotton swabs for 30 s; this was done once every 48 h, three times in total. The rats in the periodontitis + hyperoside group were given hyperoside (20 mL, 200 mg·mL^−1^) at the site of LPS injection, whereas the rats in the model group were given corn oil as control. Drug injection was repeated every other day on three separate days.

### Micro‐CT analysis

The mandible specimens were collected and scanned with micro‐CT (Siemens, Munich, Germany). The parameters we set are X‐ray source (80 kV), the 360 rotational steps (500 ms per time) and a node current (500 μA). Images were analyzed with 3D scanning software (DataViewer/CT Analyzer/CT volume).

### Hematoxylin and eosin and Masson staining assays

Tissues were fixed in 4% paraformaldehyde, embedded in paraffin and sectioned into 4 μm. Then the samples were stained with hematoxylin for 10 min and treated with eosin or Masson for 5 min at room temperature. Histological images were acquired under a light microscope (Olympus, Tokyo, Japan) at the magnification of 200×.

### ELISA assay and alkaline phosphatase activity detection

Periodontal tissues were added with PBS (pH 7.4), followed by homogenization thoroughly with a homogenizer and centrifugation for about 20 min (4 °C, 400 *g*). Supernatant was collected to analyze the release of cytokines. The levels of TNF‐α (JEB‐13718; Jin Yibai, Nanjing, China), IL‐1β (JEB‐13503; Jin Yibai), IL‐6 (JEB‐14081; Jin Yibai) and alkaline phosphatase (ALP; Jiancheng, Nanjing, China) were measured according to the manufacturer’s directions.

### Isolation and culture of BMSCs

SD rats were decapitated and killed, and then soaked in 75% alcohol for 15 min. The long bone of SD rats was taken under sterile condition, and the femoral side was removed in D‐Hank’s balanced salt solution containing 100 mg·mL^−1^ streptomycin and 100 U·mL^−1^ penicillin (Gibco, Grand Island, NE, USA). Bone marrow was washed with α‐MEM medium and collected in a 25‐cm culture bottle. Then bone marrow was blown repeatedly and slightly to disperse the cells as much as possible and put into a jacket cell incubator at 37 °C with 5% CO_2_ for a week [31].

### Identification of BMSCs

First, BMSCs were cultured and passaged normally, and the morphological characteristics of BMSCs were observed under the microscope at P0 (Original cells), P1 (First‐passage cells), P2 (Second‐passage cells) and P3 (Third‐passage cells), respectively. Moreover, isolated BMSCs were collected and counted with a density of 1 × 10^6^ per milliliter. Following that, BMSCs were incubated with corresponding antibodies, including CD34, CD45, CD73, CD90 and CD105, at 4 °C for 30 min with low‐speed shaking. Subsequently, the cells were washed with PBS three times to remove the antibodies on the cell surface. Then the cells were resuspended by adding 500 mL PBS, and the expressions of cell surface markers were detected by flow cytometer (BD Biosciences, San Jose, CA, USA) [32].

### Cell Counting Kit‐8 assay

For cell viability, BMSCs were seeded in 96‐well plates at a density of 1 × 10^4^ cells per well for 24 h and then treated with hyperoside with indicated days. Then cell viability was examined by Cell Counting Kit‐8 (CCK‐8) kit (Beyotime Biotechnology, Shanghai, China) based on the specification. The absorbance was detected at 490 nm by microplate reader (Tecan Infinite M200 Micro Plate Reader; LabX, Midland, ON, Canada).

### EdU assay

The proliferation of BMSCs treated with hyperoside was assessed by EdU assay. BMSCs (1 × 10^4^ per well) were cultured in 96‐well plates and treated with hyperoside. Then treated BMSCs were fixed with 4% paraformaldehyde, Triton X‐100 was used to permeabilize the nuclear membrane and treated BMSCs were blocked with goat serum for 1 h. Further, treated BMSCs were stained according to the manufacturer’s suggestions.

### Cell‐cycle distribution and cell apoptosis

BMSCs with the density of 1 × 10^6^ per milliliter were fixed with precooled 70% EtOH at 4 °C overnight. PBS was used to wash cells three times, and 100 μL RNase A was added to mix the solution fully for 30 min at 37 °C. Forward, 400 μL propidium iodide (PI) dye solution was added to mix well, and incubated for 30 min at 4 °C. Then, the cell‐cycle distribution was detected by flow cytometer (BD Biosciences). The role of hyperoside in the apoptosis of BMSCs was measured by the Annexin V–FITC kit (Beyotime Biotechnology). In brief, BMSCs were treated with hyperoside, harvested with trypsin, washed with PBS and resuspended in 500 μL binding buffer. Next, BMSCs were treated with 5 μL Annexin V–FITC and 10 μL PI for 15 min in the dark. Finally, apoptotic cells were determined by flow cytometer (BD Biosciences).

### Alizarin red and ALP staining assays

BMSCs were collected from each group, washed with PBS and fixed with 10% neutral formalin for 10–30 min. Then the fixed solution was removed, and BMSCs were washed twice by PBS. Further, alizarin red staining solution or ALP staining solution was added to cover the sample for 1–5 min. After that, the cells were washed twice with PBS and observed under inverted microscope. For the quantification of alizarin red, 10% cetylpyridinium chloride was used to dissolve relevant calcium composition.

### Quantitative real‐time PCR

Total RNA was separated from BMSCs and tissues by TRIzol reagent (Invitrogen, Carlsbad, CA, USA), and cDNA was synthesized by TaqMan Reverse Transcription Kit (Applied Biosystems, Foster City, CA, USA). Routine quantitative real‐time PCR was implemented using ABI 7300‐fast RT PCR system (Applied Biosystems) with SYBR Green PCR Kit (Qiagen, Hilden, Germany) based on the specifications. Relative expressions of mRNAs were evaluated by $2^{‐\Delta \Delta C_{t}}$ method with Gapdh as internal reference. The primer sequences used were listed as follows: Runx2 forward, 5′‐CATGGCCGGGAATGATGAG‐3′, and reverse, 5′‐TGTGAAGACCGTTATGGTCAAAGTG‐3′; Osx forward, 5′‐TGCCAATGACTACCCACCC‐3′, and reverse, 5′‐TGCCCACCACCTAACCAA‐3′; Alp forward, 5′‐CAGTGGTATTGTAGGTGCTGTG‐3′, and reverse, 5′‐TTTCTGCTTGAGGTTGAG GTTAC‐3′; Ocn forward, 5′‐CATGAGAGCCCTCACA‐3′, and reverse, 5′‐AGAGCGACCCTAGAC‐3′; Gapdh forward, 5′‐TGGAGTCTACTGGCGTCTT‐3′ and reverse, 5′‐TGTCATATTTCTCGTGGT TCA‐3′.

### Western blot assay

Proteins were extracted from BMSCs and tissues by radioimmunoprecipitation assay lysis buffer and quantified by BCA kit (Beyotime Biotechnology). Proteins were separated by 10% SDS/PAGE and transferred into poly(vinylidene difluoride) membranes (Millipore, Bedford, MA, USA). Then the membranes were blocked with 5% nonfat milk in TBST at room temperature for 1 h and treated with the primary antibody at 4 °C overnight. Membranes were washed with TBST three times and probed with horseradish peroxidase‐conjugated secondary antibody (1: 2000, ab6728; Abcam, Cambridge, MA, USA) for 1 h at room temperature. Finally, protein blots were visualized by enhanced chemiluminescence kit (Millipore) and quantified using imagej software (National Institutes of Health, Bethesda, MD, USA, version 4.3). The primary antibodies were as follows: RUNX2 (ab192256, 1 : 1000; Abcam, Cambridge, UK), OSX (ab22552, 1 : 1000; Abcam), ALP (ab83259, 1 : 1000; Abcam), Dentin sialophosphoprotein (DSPP) (ab216892, 1 : 1000; Abcam), p‐P65 (ab86299, 1 : 1000; Abcam), P65(ab16502, 1 : 1000; Abcam), p‐IκBα (ab133462, 1 : 1000; Abcam), IκBα (ab32518, 1 : 1000; Abcam) and (glyceraldehyde‐3 phosphate dehydrogenase) (ab181602, 1 : 1000; Abcam).

### Statistical analysis

Data were analyzed by graphpad prism 5.0 (GraphPad Software Inc., San Diego, CA, USA) and presented as mean ± SD. One‐way ANOVA followed by Tukey’s *post hoc* test was used to compare differences among multiple groups. *P* < 0.05 was indicated as statistically significant.

## Results

### Effects of hyperoside on periodontitis in rats

**Fig. 1:**
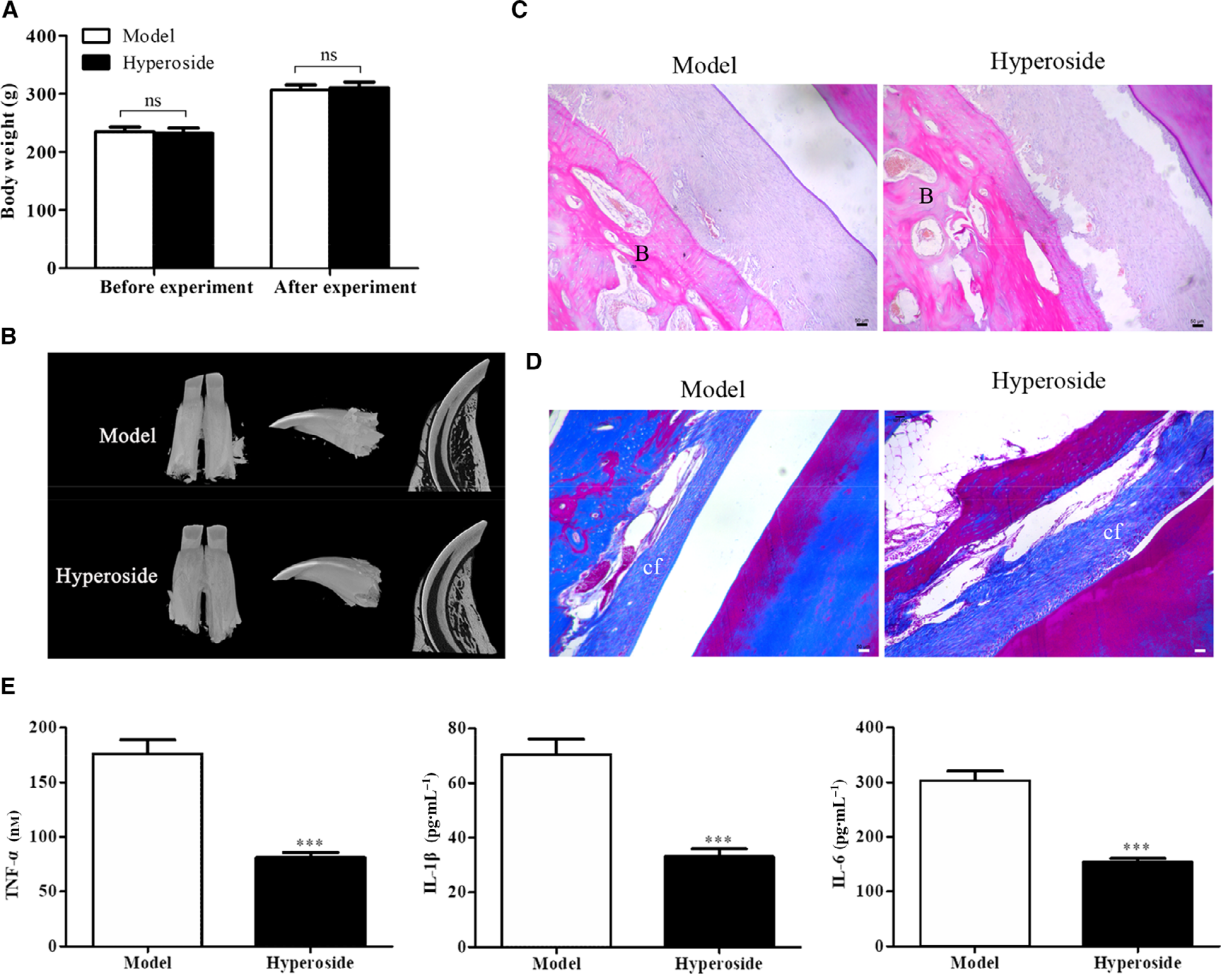
Effects of hyperoside on periodontitis in rats. (A) Body weight of rats from the model group and hyperoside group was measured before and after experiment. (B) The absorption of alveolar bone of periodontitis rats treated with hyperoside was detected with micro‐CT. (C) Periodontal tissue pathology of periodontitis rats treated with hyperoside was evaluated by hematoxylin and eosin staining assay. (D) The expression of collagen fibers in periodontitis rats treated with hyperoside was evaluated by Masson staining assay. (E) The levels of TNF‐α, IL‐1 and IL‐6 in periodontitis rats treated with hyperoside were determined by ELISA assay (*n* = 3, means ± SD). Data are representative of three independent experiments and were analyzed using ANOVA multiparametric test. ***P < 0.001. Scale bars: 50 μm. B, bone tissues; cf, collagen fibers.

To explore the possible role of hyperoside in the development and progression of periodontitis, we constructed the periodontitis rat model and treated with hyperoside. First, we measured the rat body weight before and after treatment with hyperoside, and the data of Fig. 1A showed that the body weight of rats from the model group and hyperoside group was significantly increased compared with those before treatment, but there was no significant difference between the two groups. Then micro‐CT was performed to investigate the effects of hyperoside on the absorption of alveolar bone of rats with periodontal disease, and the data of Fig. 1B indicated that in the model group, alveolar bone resorption reached one‐third of the middle root zone and the root bifurcations were completely exposed, whereas hyperoside treatment could be the absorption of alveolar bone. In addition, hematoxylin and eosin staining was used to investigate the effect of hyperoside on periodontal tissue pathology of rats with periodontal disease, and the data of Fig. 1C showed that the gingival morphology and structure of rats in the model group had disappeared, the epithelium in the sulcus was eroded and a large number of inflammatory cells were infiltrated. In contrast, after hyperoside intervention, the periodontal disease of rats was significantly improved, the gingival morphology and structure of rats were partially disappeared and the infiltration of inflammatory cells was significantly relieved. Besides, Masson staining was adapted to investigate the effects of hyperin on collagen fibers in periodontal disease rats, and the results of Fig. 1D showed that the collagen fibers in periodontal tissues of the model group were disordered and even broken, and after hyperoside intervention, the density of collagen fibers in periodontal tissue was uniform and arranged orderly. Finally, ELISA was carried out to evaluate the effects of hyperoside on the production of proinflammation factors in periodontitis rats, and the data of Fig. 1E demonstrated that the production of TNF‐α, IL‐1β and IL‐6 was overexpressed in periodontitis rats, whereas hyperoside treatment could notably inhibit the production of TNF‐α, IL‐1β and IL‐6. These data suggested that hyperoside ameliorated periodontitis in rats.

### Isolation and identification of rat BMSCs

**Fig. 2:**
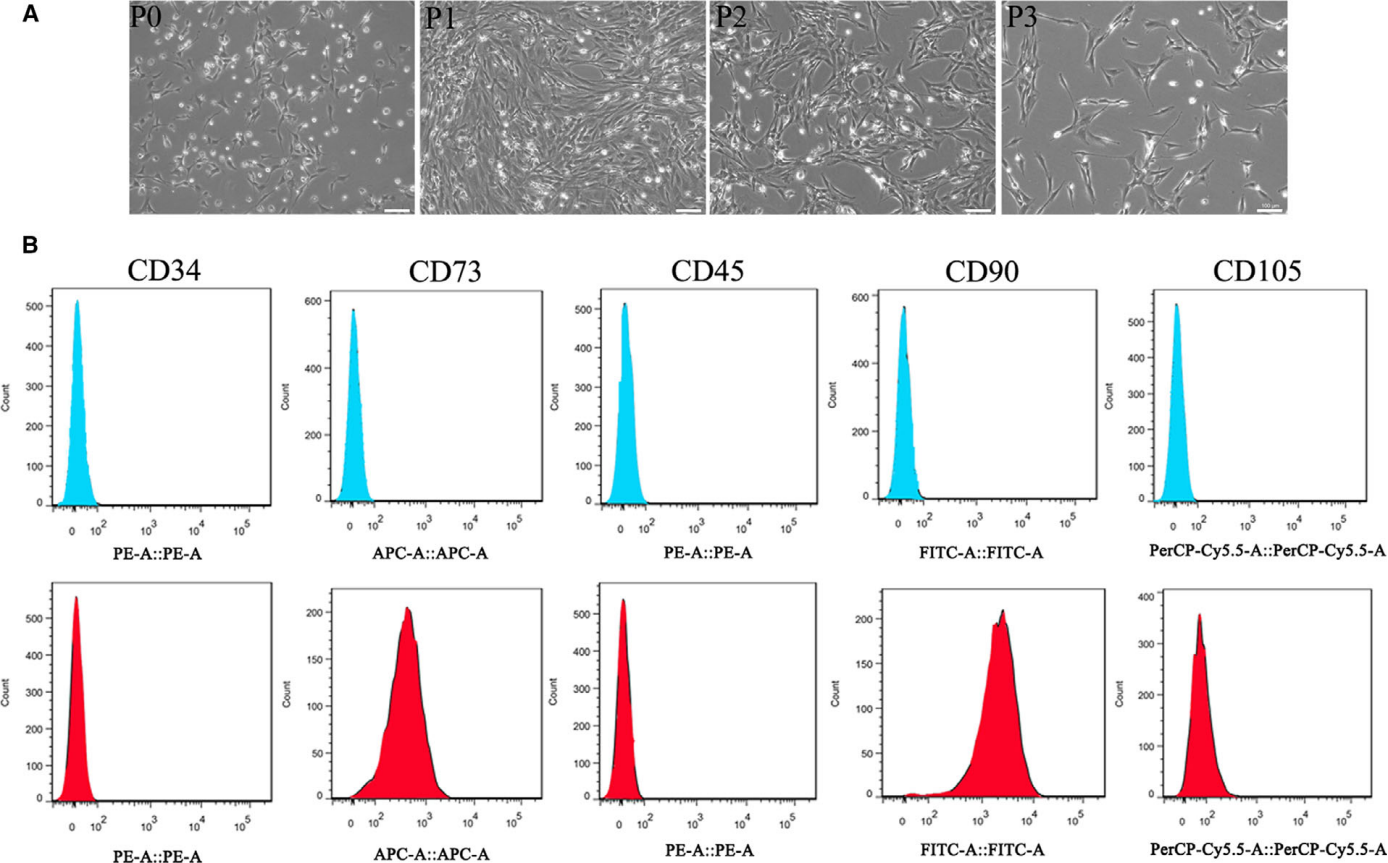
Isolation and identification of rBMSCs. (A) Morphological observation of rBMSCs of P0‐P3. (B) Flow cytometry analysis showed that rBMSCs were positive for CD73, CD90, CD105, CD34 and negative for CD45. Scale bars: 100 μm. rBMSCs, rat bone mesenchymal stem cells; CD, cluster differentiation

To explore the underlying mechanisms of hyperoside on the development and progression of periodontitis, first, we isolated rat bone mesenchymal stem cells (rBMSCs) from the SD rats, which were further identified with morphological observation and flow cytometry analysis. As shown in Fig. 2A, at the beginning, the cells were suspended in the medium, round and different in size. Then the cells began to adhere to the wall, and the cells were long fusiform and spindle shaped after culture for 6 h. After 48 h of culture, adherent cells were increased and a small number of cell colonies formed. After 7 days, a large number of cells were spindle or spindle shaped, and the number of colonies was increased significantly. After 14 days, the cells were laid on the whole cell culture plate, regular cobblestone‐like. After that time, the cell proliferation was active and the morphology could not be changed significantly. In addition, the data of Fig. 2B indicated that CD73, CD90 and CD105 were positively expressed, whereas CD34 and CD45 were negatively expressed in isolated cells. These results confirmed that the cells isolated from SD rats were rBMSCs.

### Effects of hyperoside on viability, proliferation, apoptosis and cell‐cycle distribution in rBMSCs

**Fig. 3:**
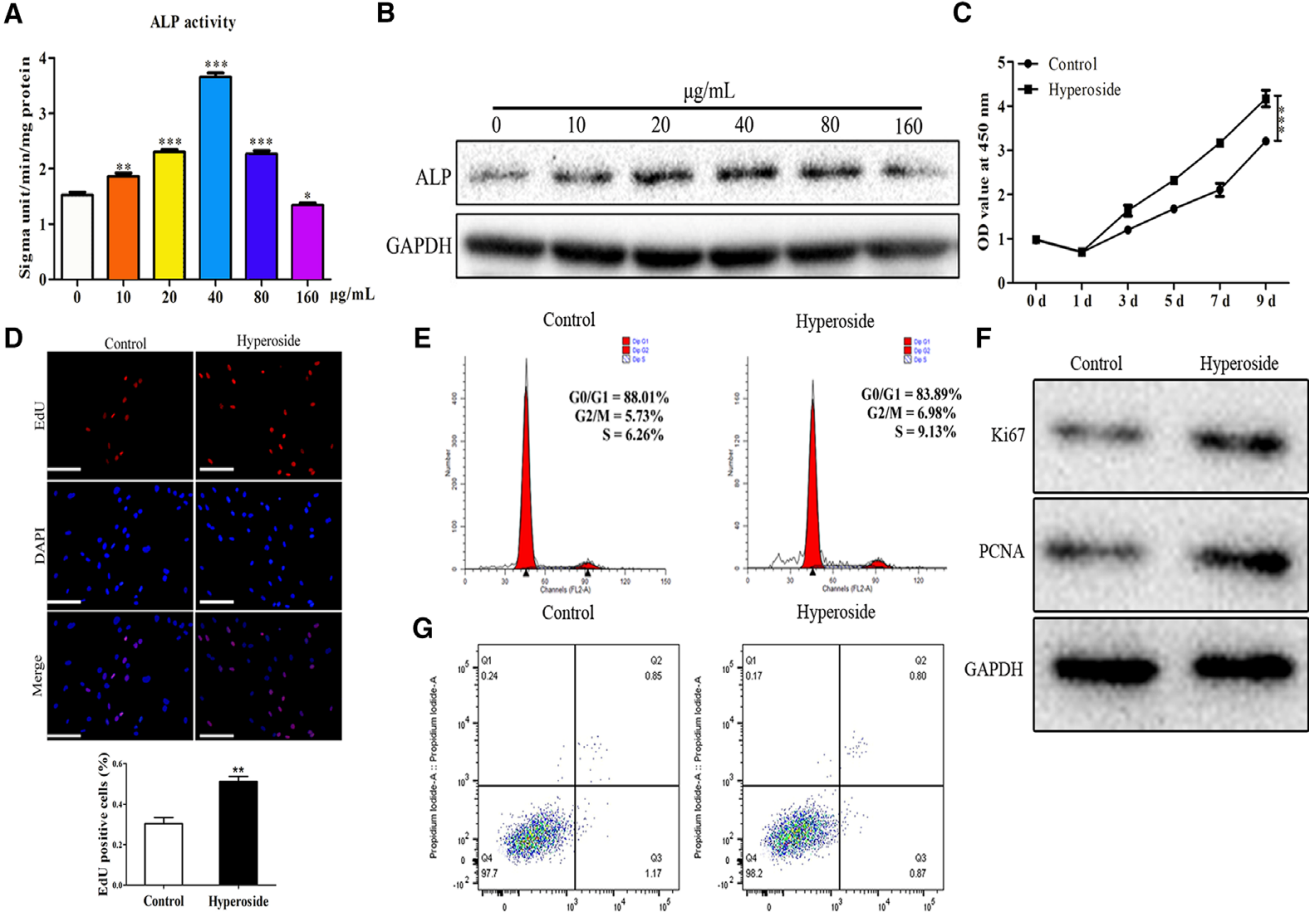
Effects of hyperoside on viability, proliferation, apoptosis and cycle distribution in rBMSCs. (A) ALP activity was performed to evaluate the effects of hyperoside with different concentrations on the activity of ALP in rBMSCs. (B) Western blot assay was performed to evaluate the effects of hyperoside with different concentrations on the expression of ALP protein in rBMSCs. (C) CCK‐8 assay performed that the viability of rBMSCs was obviously enhanced in the hyperoside group compared with the control group. (D) EdU assay was performed to evaluate the effects of hyperoside on proliferation in rBMSCs. (E) Flow cytometry assay was performed to evaluate the effects of hyperoside on cell‐cycle distribution in rBMSCs. (F) Western blot assay was performed to evaluate the effects of hyperoside on the protein expressions of Ki67 and PCNA in rBMSCs. (G) Flow cytometry assay was performed to evaluate the effects of hyperoside on apoptosis in rBMSCs (*n* = 3, means ± SD). Data are representative of three independent experiments and were analyzed using ANOVA multiparametric test. *P < 0.05, **P < 0.01, ***P < 0.001, compared with that of control group. Scale bars: 200 μm.

To investigate the effects of hyperoside on the functions of rBMSCs, first, ALP activity and western blot were performed to evaluate the effects of hyperoside on the expression of ALP, and the data of Fig. 3A,B showed that ALP was highest expressed when the concentration of hyperoside was 40 μg·mL^−1^. Therefore, hyperoside with the concentration of 40 μg·mL^−1^ was chosen for further study. Thus, to evaluate whether hyperoside could affect the proliferation of rBMSCs, first, we performed CCK‐8 assay to evaluate the effects of hyperoside on viability of rBMSCs, and the data of Fig. 3C indicated that after treatment with hyperoside, the viability of rBMSCs was obviously enhanced. Besides, EdU assay was adapted to evaluate the suppressive effects of hyperoside on proliferation in rBMSCs. As shown in Fig. 3D, hyperoside could significantly up‐regulate the EdU‐positive cells in rBMSCs. In addition, the effects of hyperoside on cell‐cycle distribution of rBMSCs were evaluated by flow cytometry assay, and the results of Fig. 3E suggested after the intervention of hyperoside, the distribution of rBMSCs in G1 phase was decreased significantly. Further, western blot was used to detect the protein expression levels related to proliferation, including Ki67 and PCNA, and the data of Fig. 3F showed that compared with the blank control group, the expression level of Ki67 and PCNA in the hyperoside intervention group was significantly higher. Moreover, the effects of hyperoside on apoptosis of rBMSCs were evaluated by flow cytometry assay, and the results of Fig. 3G suggested hyperoside had no significant impact on the apoptosis of rBMSCs. These data suggested that hyperoside promoted proliferation in rBMSCs.

### Effects of hyperoside on osteogenic differentiation both *in vivo* and *in vitro*

**Fig. 4:**
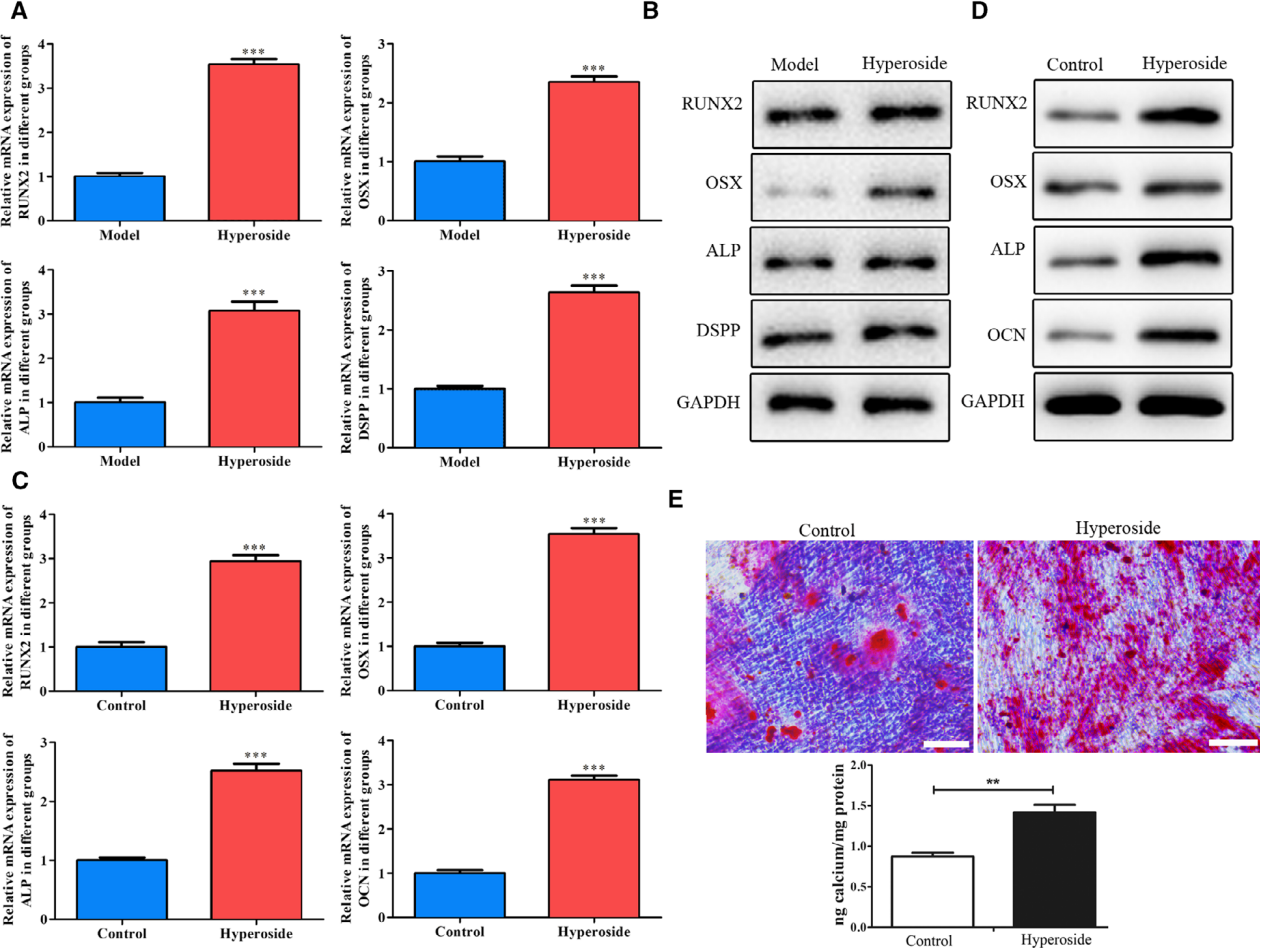
Effects of hyperoside on osteogenic differentiation both *in vivo* and *in vitro*. (A, B) Quantitative RT‐PCR (A) and western blot assays (B) were performed to evaluate the mRNA and protein expressions of RUNX2, OSX, ALP and DSPP in periodontitis rats treated with hyperoside. (C, D) Quantitative RT‐PCR (C) and western blot (D) were carried out to detect the effects of hyperoside on mRNA and protein expressions of RUNX2, OSX, ALP and OCN in rBMSCs. (E) Alizarin red staining was used to investigate the effects of hyperoside on the mineralization in rBMSCs (*n* = 3, means ± SD). Data are representative of three independent experiments and were analyzed using ANOVA multiparametric test.**P < 0.01, ***P < 0.001 compared with that of the model group or control group. Scale bars: 200 μm.

In the development of periodontitis, the balance between osteoblasts and osteoclasts plays an important role. First, we collected periodontal tissues, and quantitative RT‐PCR and western blot were performed to evaluate the mRNA and protein expressions of RUNX2, OSX, ALP and DSPP, and the data of Fig. 4A,B showed that, compared with the model group, hyperoside could significantly promote the mRNA and protein expressions of RUNX2, OSX, ALP and DSPP. Further, quantitative RT‐PCR and western blot were carried out to detect the effects of hyperoside on the expressions of osteogenic differentiation‐related indexes, including RUNX2, OSX, ALP and OCN in rBMSCs, and the data of Fig. 4C,D suggested that hyperoside significantly promoted the mRNA and protein expressions of RUNX2, OSX, ALP and OCN. Further, alizarin red staining was used to investigate the effects of hyperoside on the mineralization of rBMSCs, and the results of Fig. 4E showed that calcium deposition was produced in rBMSCs after hyperoside intervention. These data suggested that hyperoside promoted osteogenic differentiation both *in vivo* and *in vitro*.

### **Effects of hyperoside on the expression of the NF‐κB signaling pathway both** *in vivo* **and *in vitro***

**Fig. 5:**
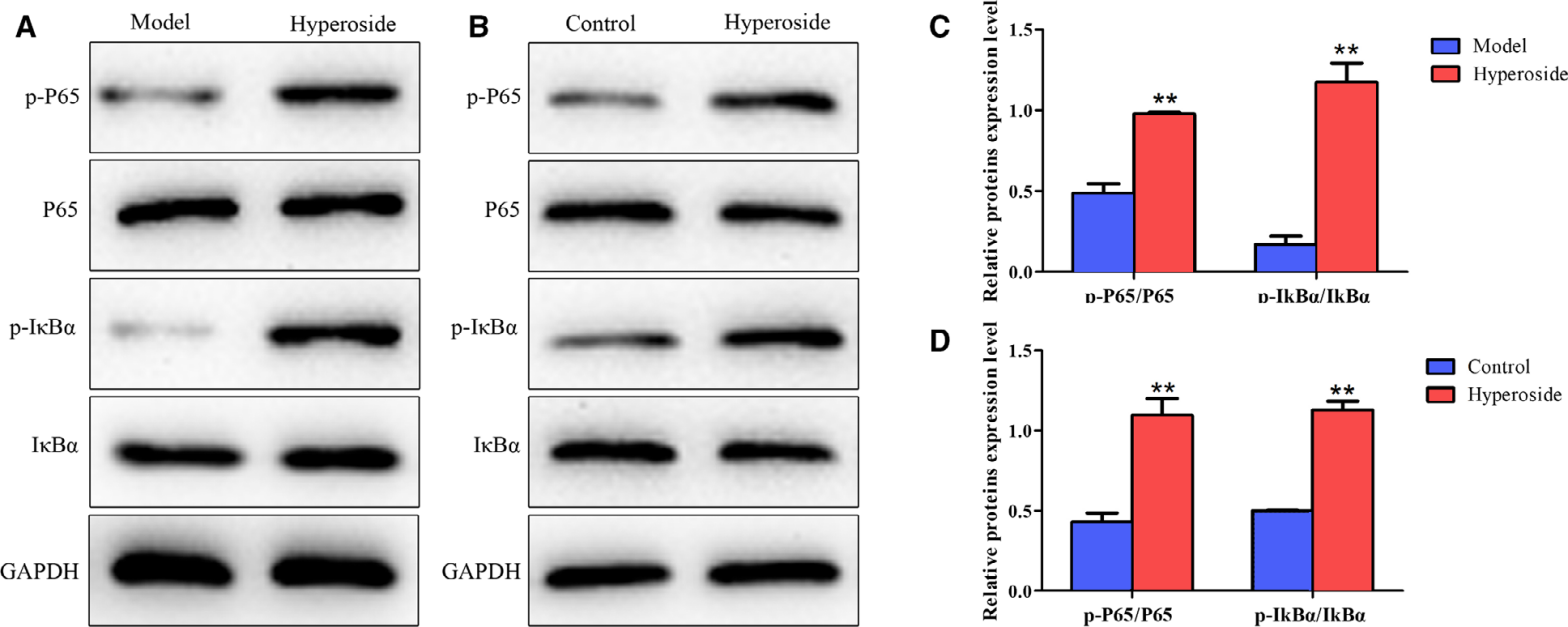
Effects of hyperoside on the expression of the NF‐κB signaling pathway both *in vivo* and *in vitro*. Western blot was performed to evaluate the effects of hyperoside on the expressions of the NF‐κB signaling pathway both (A) *in vivo* and (B) *in vitro*. (C, D) Histograms showed the quantification of band intensities in (A) and (B), respectively (*n* = 3, means ± SD). Data are representative of three independent experiments and were analyzed using ANOVA multiparametric test. **P < 0.001.

The NF‐κB signaling pathway, as one of the main signal transduction pathways, exhibited an essential role in the development and progression of periodontitis. First, western blot was performed to evaluate the effects of hyperoside on the expressions of the NF‐κB signaling pathway *in vivo*, and the data of Fig. 5A,C showed that, compared with the model group, hyperoside could significantly promote the expressions of the NF‐κB signaling pathway. Similarly, western blot was carried out to detect the effects of hyperoside on the expressions of the NF‐κB signaling pathway *in vitro*, and the data of Fig. 5B,D showed that hyperoside could significantly promote the expressions of the NF‐κB signaling pathway. These data suggested that hyperoside activated the NF‐κB signaling pathway both *in vivo* and *in vitro*.

### Effects of hyperoside on biological functions in rBMSCs mediated by the NF‐κB signaling pathway

**Fig. 6:**
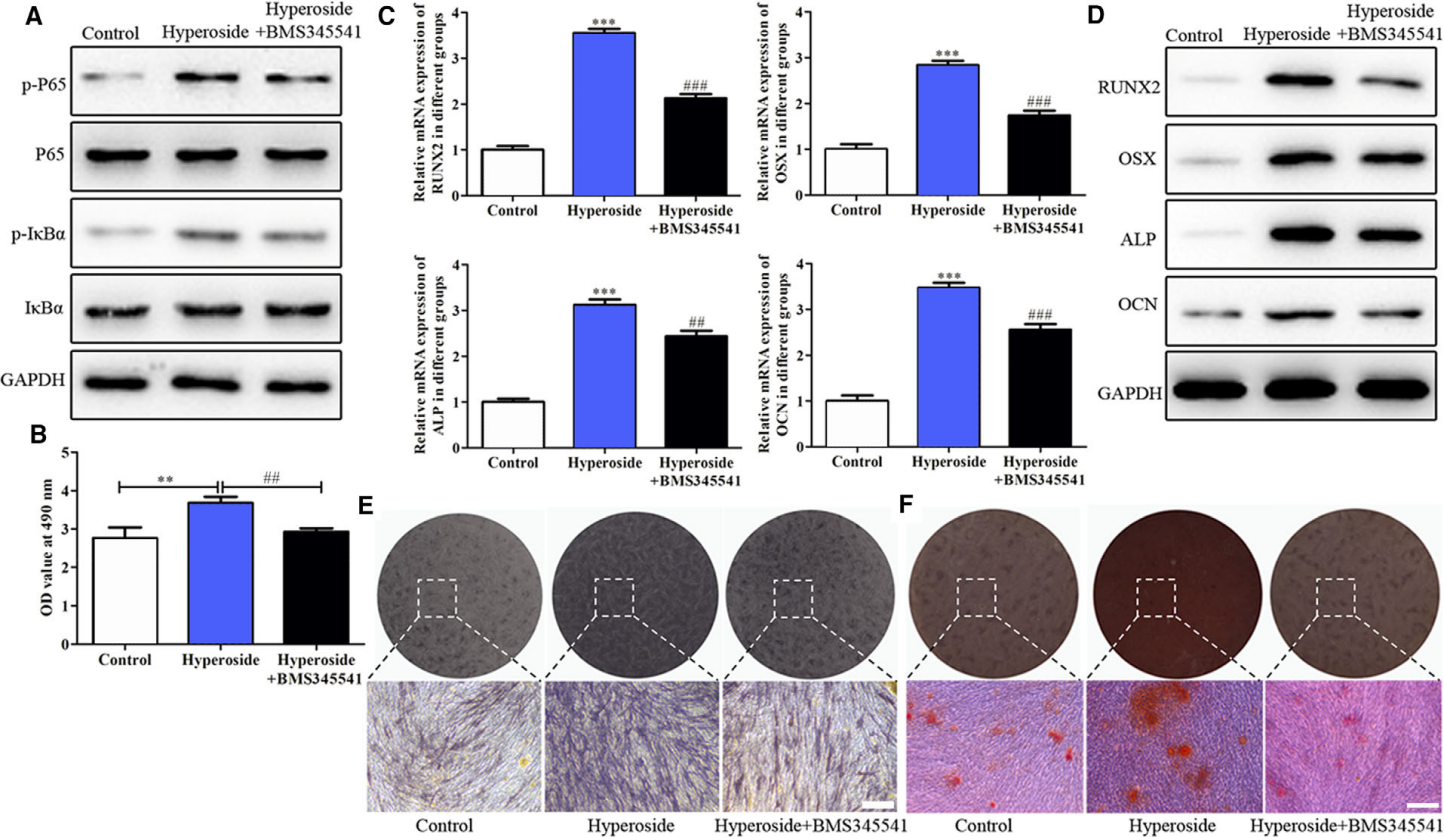
Effects of hyperoside on biological functions in rBMSCs mediated by the NF‐κB signaling pathway. (A) Western blot was performed to evaluate the effects of BMS345541 on the expressions of the NF‐κB signaling pathway in rBMSCs. (B) CCK‐8 assay showed that BMS345541 reversed the effects of hyperoside on the viability of rBMSCs. (C, D) Quantitative RT‐PCR (C) and western blot (D) were carried out to detect the effects of BMS345541 on mRNA and protein expressions of RUNX2, OSX, ALP and OCN in rBMSCs. (E) ALP staining was performed to evaluate the effects of BMS345541 on osteogenic differentiation in rBMSCs. (F) Alizarin red staining indicated that BMS345541 restored the effects of hyperoside on the cell mineralization of rBMSCs (*n* = 3, means ± SD). Data are representative of three independent experiments and were analyzed using ANOVA multiparametric test. **P < 0.01, ***P < 0.001 compared with that of the control group; ^*##*^ *P* < 0.01, *^###^* *P* < 0.001 compared with that of the hyperoside group. Scale bars: 200 μm.

Further, to explore whether the NF‐κB signaling pathway was involved in the effects of hyperoside on the biological functions in rBMSCs, we chose BMS345541, a NF‐κB signaling pathway inhibitor, to inhibit the NF‐κB signaling pathway in rBMSCs treated with hyperoside. As shown in Fig. 6A, BMS345541 could inhibit the expressions of the NF‐κB signaling pathway activated by hyperoside. Then the results of CCK‐8 showed that BMS345541 reversed the effects of hyperoside on the viability of rBMSCs shown in Fig. 6B. The results of Fig. 6C,D revealed that BMS345541 restored the effects of hyperoside on the osteogenic differentiation‐related indexes detected by quantitative RT‐PCR and western blot analysis. The data of Fig. 6E,F indicated that BMS345541 restored the effects of hyperoside on the osteogenic differentiation and cell mineralization detected with ALP and alizarin red staining assays. These data suggested that hyperoside promoted proliferation and osteogenic differentiation in rBMSCs mediated by the NF‐κB signaling pathway.

## Discussion

At present, some drugs, which have been widely used for the treatment of periodontitis clinically, have a certain toxic side effect, such as changes of oral microflora, further leading to tooth discoloration. In addition, they can cause gastrointestinal disorders, usually accompanied by diarrhea, nausea and vomiting, or rarely phototoxicity and accumulation in bones and teeth [33, 34]. Based on these, a large number of studies have shown that the application of Chinese herbal medicine is a new trend in the prevention and treatment of periodontitis, with fewer adverse effects on patients. For example, paeonol exhibited protective effects against periodontitis through regulation of the Nrf2/NF‐κB/NFATc1 signaling pathway [35]. Standardized *Boesenbergia pandurata* extract and its active compound panduratin A could obviously improve LPS‐induced periodontal inflammation and alveolar bone loss in rats [36]. Moreover, mangiferin significantly ameliorated *Porphyromonas gingivalis*‐induced experimental periodontitis by regulating NF‐κB and JAK1–STAT1/3 signaling pathway [37]. Therefore, more and more studies on natural products or phytochemicals are expected to become acceptable substitutes or supplements for the treatment of periodontitis. Hyperoside is a kind of traditional Chinese medicine with anti‐inflammatory, immunoregulatory and antioxidant activities, and hyperoside can play a role by regulating the transmission of inflammatory factors, immune molecules and related signal pathways, which has been widely used in clinic [27, 28, 29]. Therefore, this study was designed to investigate the possible role and underlying mechanisms of hyperoside in the development and progression of periodontitis, and the *in vivo* study results showed that hyperoside obviously ameliorated periodontitis achieved by improving absorption of alveolar bone, inflammatory infiltration and collagen fibers. These data were similar to previous studies, which further confirmed the positive effects of hyperoside on periodontitis.

Periodontal therapy aims to achieve complete regeneration of these structures, especially alveolar bone. So far, several methods have been used to achieve this goal, such as the use of various bone grafts, growth factors and barrier membranes [38]. The regeneration of alveolar bone depends on the differentiation of BMSCs or periodontal ligament stem cells into osteoblasts, and osteoblasts are the key to the formation of new bones. Inducing and activating more osteoblasts can promote the regeneration of alveolar bone [39]. BMSCs have a strong ability of self‐renewal, so that they can produce the same cells as themselves in a long period of time. Studies have shown that some aging BMSCs still have strong proliferation ability, even at 80 ℃ [40]. Cell proliferation is related to many factors, not only the influence of cytokines but also the culture matrix and cell density. BMSCs can obtain higher amplification at low cell density (1.5–3 per cm^2^), whereas at high density (12 per cm^2^), the results were just the opposite. These experimental results showed that the self‐renewal ability of BMSCs *in vivo* and *in vitro* may not be the same [41]. Although BMSCs have a wide range of proliferation and differentiation ability *in vitro*, BMSCs are generally in a static state *in vivo*. Only specific environmental changes or external stimuli, such as damage and tissue degeneration, can activate BMSC proliferation and directional differentiation signal transduction [42, 43]. As expected, in our study, we found that hyperoside can promote the proliferation of BMSCs.

Multidifferentiation potential is considered to be an important biological feature of BMSCs. *In vitro* experiments showed that BMSCs can be differentiated into many known cell lineages under appropriate induction conditions, such as osteoblasts, adipocytes, chondrocytes, cardiomyocytes and so on [44, 45]. The process of osteogenesis *in vitro* has four stages: transformation, cell proliferation, cell aggregation and secretion, and extracellular matrix calcification. Maniatopoulos *et al*. [46] reported for the first time that BMSCs obtained from bone marrow of adult rats can form calcified bonelike tissue *in vitro*, and X‐ray diffraction analysis confirmed that the calcified tissues have a hydroxyapatite‐like structure, which proved that BMSCs cultured *in vitro* can differentiate into osteoblasts. Ouyang *et al*. [47] reported that BMSCs grew in sheet shape and arranged closely after adding ascorbic acid into the culture medium. In addition, BMSCs were implanted into the damaged site by combining with the grafted bone, and after 3 weeks, the structure of the implant was similar to that of the normal periosteum, and osteogenic differentiation was observed by morphological, histological and immunohistochemical methods [47]. Interestingly, hyperoside could promote osteogenic differentiation of BMSCs. Similarly, *in vivo* studies showed that hyperoside could promote osteogenic differentiation of periodontal tissues.

NF‐κB, as an excellent aggregation point of various signal transduction pathways, plays an important role in immune response, inflammatory response, cell proliferation and apoptosis. Sustained or overexpressed NF‐κB is closely related to the occurrence and development of inflammatory diseases. The expressions of NF‐κB transcription factor (p50/p65) and IκB in the nucleus and cytoplasm of healthy patients and periodontal tissues were detected by Huang *et al*. [48], and it has been found that the activation rate of NF‐κB in periodontal tissues (75.90%) was significantly higher than that in normal tissues (5.30%), whereas the expression of IκB periodontal tissues (5%) was significantly lower than that in normal tissues (50%), showing that NF‐κB played an important role in periodontitis. It has been demonstrated that dietary flavonoid hyperoside may have potential as a therapeutic agent for NF‐κB activation [49]. In the study of IL‐1β‐induced inflammatory mediators and NF‐κB‐induced effects on human gingival fibroblasts (HGFs), Vardar‐Sengul *et al*. [50] found that the gene expression changes induced by IL‐1β were consistent with the pathological changes of periodontitis, including the increase of inflammatory factors, driving factors, transcription factors, matrix metalloproteinases and adhesion molecules, especially the increase of NF‐κB‐dependent antiapoptotic factors. Therefore, activation of NF‐κB in periodontitis can prevent apoptosis and decrease HGF activity, indicating that NF‐κB played a role in HGF [50]. In this study, we found that hyperoside promoted the expressions of the NF‐κB signaling pathway both *in vivo* and *in vitro*. To further verify role of the NF‐κB signaling pathway in occurrence and development of periodontitis, we chose a NF‐κB signaling pathway inhibitor, BMS345541, to inhibit the NF‐κB signaling pathway, and we found that BMS345541 reversed the effects of hyperoside on the biological functions of BMSCs.

In conclusion, we confirmed that hyperoside exhibited potential therapeutic properties against periodontitis by promoting proliferation and osteogenic differentiation of BMSCs by activation of the NF‐κB signaling pathway. Therefore, our study provides evidence that hyperoside may emerge as a therapeutic option for periodontitis treatment.

## Conflict of interest

The authors declare no conflict of interest.

## Author contributions

TX and XW conceived and designed the study, collected and assembled data, and wrote the manuscript; these two authors contributed equally to this work. YY and ZZ performed data analysis and interpretation. CY and NZ reviewed the data. JY conceived and designed the study, provided financial support and study material, performed data analysis and interpretation, and approved the final version of the manuscript. All authors read and approved the manuscript.

## Data Availability

The authors state that all data will be available from the corresponding author upon reasonable request.
